# Short-term effects of Mediterranean diet on nutritional status in adults affected by Osteogenesis Imperfecta: a pilot study

**DOI:** 10.1186/s13023-024-03100-5

**Published:** 2024-03-01

**Authors:** Ramona De Amicis, Simona Bertoli, Amalia Bruno, Giulia De Carlo, Alberto Battezzati, Andrea Foppiani, Alessandro Leone, Antonella Lo Mauro

**Affiliations:** 1 International Center for the Assessment of Nutritional Status and the Development of Dietary Intervention Strategies (ICANS-DIS), Department of Food, Environmental and Nutritional Sciences (DeFENS), University of Milan, 20133 Milan, Italy; 2 IRCCS Istituto Auxologico Italiano, Obesity Unit and Laboratory of Nutrition and Obesity Research, Department of Endocrine and Metabolic Diseases, 20145 Milan, Italy; 3 IRCCS Istituto Auxologico Italiano, Clinical Nutrition Unit, Department of Endocrine and Metabolic Medicine, 20100 Milan, Italy; 4 Dipartimento di Elettronica, Informazione e Bioingegneria, Politecnico di Milano, Piazza, Leonardo Da Vinci, 20133 Milan, Italy

**Keywords:** Osteogenesis Imperfecta, Mediterranean diet, Nutritional status, Body composition, Dietary treatment

## Abstract

**Background:**

Osteogenesis Imperfecta (OI) is a heterogeneous group of connective tissue disorders, characterized by varying degrees of skeletal fragility. Patients experience a range of comorbidities, such as obesity, cardiovascular, and gastrointestinal complications, especially in adulthood. All aspects that could benefit from dietary intervention. The aim of this study was to evaluate the effects of a 6-months restricted Mediterranean Diet (rMD) on nutritional status in adult patients affected by OI.

We carried out a 6-months longitudinal pilot study. 14 adults (median age: 35 years; 7 women; 7 OI type III) where recruited in 2019 among the members of As.It.O.I., the Italian Association of Osteogenesis Imperfecta. As.It.O.I. All the evaluations were performed at the University of Milan, Italy. The rMD provided a reduction of 30% from daily total energy expenditure. 45% of calories derived from carbohydrates, 35% from fat and 0.7–1.0 g/kg of body weight from proteins. Comparisons of continuous variables after 6 months of intervention were performed by the paired t-test. All P-values were two-tailed, and *p* < 0.05 was considered significant.

**Results:**

Patients showed significant improvement in anthropometric measurements (BMI = 30.5 vs 28.1 kg/cm^2^, *p* < 0.001; Body Fat % = 32.9 vs 29.9, *p* = 0.006; Waist circumferences = 83.6 vs 79.6 cm; *p* < 0.001; Arm Fat Area = 29.8 vs 23.07 cm^2^; *p* < 0.011) and energy expenditure (REE/kg = 27.2 vs 29.2 kcal/kg, *p* < 0.001). Glucose and lipid profiles improved (Δglycemia = − 8.6 ± 7.3 mg/dL, *p* = 0.003; ΔTC = − 14.6 ± 20.1 mg/dL, *p* = 0.036; ΔLDL = − 12.0 ± 12.1 mg/dL, *p* = 0.009). Adherence to the MD significantly increased, moving from a moderate to a strong adherence and reporting an increased consumption of white meat, legumes, fish, nuts, fruits and vegetables.

**Conclusion:**

A rMD was effective in improving nutritional status and dietary quality in adults with OI. These results underscores the need to raise awareness of nutrition as part of the multidisciplinary treatment of this disease.

## Introduction

The term Osteogenesis Imperfecta (OI) refers to a group of inherited connective tissue disorders, a heterogeneous group of diseases characterised by varying degrees of skeletal fragility and deformity, decreased bone mass and susceptibility to bone fractures [1]. In most cases, OI is due to mutations in the COL1A1 and COL1A2 genes, which code for the α-1 and α-2 chains of type I collagen, the main component of bones, muscles and ligaments. In OI, these genetic defects can either reduce the amount of collagen (quantitative defects) or its structure (qualitative defects); in the former case, we have a milder phenotype and, in the latter a severe phenotype [1]. At the clinical level, five forms of OI have been identified. The most prominent clinical sign in all types of OI is skeletal fragility, manifested by multiple fractures. OI type 2 is lethal, type 3 is severe, types 4 and 5 are moderate, and type 1 is mild [1].

In addition to bone fragility, with a high incidence of fractures and skeletal deformities, other manifestations of the disease are dentinogenesis imperfecta, joint hypermobility, cardiovascular complications, muscle weakness, and respiratory problems, which vary according to thoracic and/or spinal deformities [2–4]. In patients with OI, it is not uncommon to also find constipation, especially in the most severe forms: pelvic asymmetry and limited mobility are among the main causes of constipation. Oral problems due to dentinogenesis imperfecta (with reduced muscle strength and tongue control) can lead to difficulties in chewing hard and solid foods, reducing dietary intake and promoting undernutrition. In some individuals with the most severe forms, there may also be difficulties in swallowing [5]. By contrast, the abnormal reduction of physical activity leads to excess weight and its related problems, such as type II diabetes, hypertension and dyslipidemia, not uncommon in patients with OI [6]. In one study Chagas et al. [7] found an increase in fractures and bone disorders in women with increased fat mass, independent of other parameters such as weight, age or level of physical activity. In our recent study, we showed how, in adulthood, abnormalities of nutritional status cause respiratory and sleep-related complications such as obstructive sleep apnea, with a significant reduction in quality of life [3].

All these aspects could benefit from a dietary intervention [7]. Diet is a modifiable factor in the development and maintenance of health, including bone mass. Current research shows that the consumption of food groups typical of the Mediterranean diet (MD), such as fruit, vegetables, nuts, low-fat dairy products, and fish, is essential for maintaining good bone health due to the synergistic effect of nutrients such as fiber, protein, vitamins and minerals [8]. Moreover, the use of extra virgin olive oil as the primary source of fat has been shown to be beneficial in preventing bone loss, especially during a weight-loss program, probably due to the high content of polyphenols. MD is also a diet poor in noxious foods, and moderate alcohol consumption that could have detrimental effects on bone mass, disrupting the balance of vitamins and local growth factors that regulates the distribution of calcium between blood and bone by affecting the hormones that regulate calcium metabolism as well as the hormones that influence calcium metabolism indirectly [9, 10]. Moreover, salt loading resulted in urinary calcium loss and increased bone turnover [11]. So, MD may positively interact in this crosstalk between bone-muscle-adipose tissue; however, surprisingly, there is no study on the effect of a protective dietary pattern in the management of nutritional abnormalities in OI, but clinical studies are focusing only on the role of calcium intake and, what's more, only on paediatric patients [7, 12–14].

Therefore, the aim of this study was to evaluate the short-term effects of a MD in the nutritional status of adult patients affected by OI.

## Materials and methods

### Ethics

The study was conducted according to the statement of the Declaration of Helsinki and the use of data for publication was approved by the Ethics Board of Politecnico di Milano, Italy (Parere n. 47/2021). All patients provided written informed consent before the beginning of the study.

### Study design and inclusion criteria

This was a prospective, 6-months longitudinal pilot study of patients with OI treated with MD. The primary outcome was to evaluate the change from pre-intervention in nutritional status, considering anthropometric measurements, body composition, resting energy expenditure, and biochemical parameters, such as glucose, lipid profile (triglycerides [TG], total cholesterol [TC], low-density lipoprotein cholesterol [LDL-C], and high-density lipoprotein cholesterol [HDL-C]), liver enzymes (alanine aminotransferase [ALT], aspartate aminotransferase [AST], gamma-glutamyl transferase [GGT]), uric acid, urea and creatinine.

We conducted assessments during pre-intervention and six months of intervention.

Inclusion criteria were confirmed diagnosis of OI type III and IV, stable condition, absence of severe cardiorespiratory pathologies, willingness to participate in the study and to travel to Milan for the tests.

### Participants

Patients were recruited among the members of As.It.O.I., the Italian Association of Osteogenesis Imperfecta. As.It.O.I., by posting an announcement on the association’s Facebook page from February 2019. Nutritional measurements and diets were performed at the International Center for the Assessment of Nutritional Status (ICANS) of the University of Milan.

Forty-five patients answered the announcement willing to participate in the study, but 18 did not meet the inclusion criteria or declined to participate, with logistic and independent travelling to Milan being the principal impediment. 27 OI patients (70% women; 14 type III) were therefore recruited for this study.

### Study design

At pre-intervention, each patient filled out a 7-days dietary record elaborated by a specialized dietician, to assess habitual caloric intake, intolerances and food preferences, and to compare with the recommendation [15]. Moreover, the following variables were collected: adherence to the MD, parameters of nutritional status (anthropometric measurements, resting energy expenditure) and biochemical variables.

At follow-up, after 6-months, the same variables were collected.

The main outcome was the change of the nutritional status in terms of anthropometric parameters (body weight, waist circumference, body fat mass e fat free mass).

### Adherence to the Mediterranean diet

Adherence to the traditional MD was assessed using a validated 14-item questionnaire [16]. The guidelines set out by the Prevención con Dieta Mediterránea (PREDIMED) study group (www.predimed.es) with some adaptation already employed in previous studies were used to obtain the a MEDiterranean Diet Adherence Score (MEDAS) [17, 18]. One point was attributed for each of the following: (1) olive oil as the main cooking fat; (2) olive oil ≥ 4 tablespoons/day; (3) vegetables > 2 servings/day (or ≥ 1 portion raw or salad); (4) fruit ≥ 3 servings/day; (5) red or processed meat < 1 serving/day; (6) butter or cream or margarine < 1/day; (7) sugar-sweetened beverages < 1/day; (8) wine ≥ 3 glasses/week; (9) legumes ≥ 3 servings/week; (10) fish/seafood ≥ 3 servings/week; (11) commercial sweets and confectionery < 3/week; (12) nuts ≥ 1/week; (13) white more than red meats (yes); and (14) use of sofrito ≥ 2/week. Participants with MEDAS ≥ 9 points were considered as complying with a dietary pattern in accordance with the MD [Schroeder].

### Assessment of nutritional status

The assessment of nutritional status results from anthropometric measurements, body composition in terms of fat and fat-free mass and resting energy expenditure.

Anthropometric measurements were performed by the same trained dietitian according to conventional measurement criteria and procedures [19, 20].

Body weight (BW, kg) and body height (BH, cm) were measured with an accuracy of 100 g and 0.5 cm, respectively. Body mass index (BMI) was calculated using the formula BW (kg)/BH^2^ (m^2^). Normal weight was defined as BMI between 18.5 and 25 kg/m^2^ [21]. Waist circumference (WC) was measured in supine position at the end of a normal exhalation with an inextensible tape, with millimetre precision. The measurement was taken at the midpoint between the last rib and the iliac crest in a horizontal plane. WC was quantified with reference tables for age and sex [21]. The neck circumference (NC) was measured with the subject looking straight ahead, with shoulders slumped but not hunched. The measurement was taken at the middle of the neck, between the mid-cervical spine and the anterior half of the neck, to the nearest 1 mm, using a nonelastic plastic tape. Skinfold thickness was measured on the non-dominant side of the body with a Holtain LTD calliper at the tricipital repere site. Biceps, triceps, sub-scapular, and supra-iliac skinfolds were taken in triplicate for all sites, and the mean of the three values was calculated. Intra-observer variation for skinfold measurements ranged from 2.5% to 2.9%. Body density was calculated by the Durnin and Womersley method [22] and fat mass percentage of body weight (FM%) by the Siri formula [23]. Arm Muscular Area (AMA) and Arm Fat Area (AFA), indicators of muscle and fat depots, were also calculated and interpreted according to the NHANES percentiles [24].

### Resting energy expenditure

To measure oxygen consumption (VO_2_) and carbon dioxide production (VCO_2_) we used an open-circuit ventilated-hood system (Q-NRG, Cosmed, Roma, Italy). We took the measurements in a thermoneutral environment (ambient temperature 24–26 °C) devoid of external stimuli. At the beginning of each test, the calorimeter was calibrated: there were two reference gas mixtures (26% O_2_ and 74% N_2_; 16% O_2_, 4.09% CO_2_ and 79.91% N_2_, respectively). Subjects were fasted for at least 12 h. Data collection time was at least 20 min, with a 5 min run-in time for stabilization and time to allow to get used to the canopy and instrument noise. Steady state was determined by five consecutive minutes in which VO_2_ and VCO_2_ variations were less than 10%. Averaging the steady state values allowed the determination of 24 h Resting Energy Expenditure (REE), done by using the abbreviated Weir equation [25]. The ratio VCO_2_/VO_2_ gave the respiratory quotient (RQ). Measured REE values were compared with predicted REE value (pREE) based on the Harris-Benedict equation [26].

### Biochemical parameters

Fasting blood samples were collected by venipuncture of the antecubital vein in a sitting or reclining position, using vacuum tubes. After centrifugation (800 g for 10 min at 5 °C), aliquots of samples were stored at 80 °C until further analysis. An autoanalyzer (Cobas Integra 400 plus, Roche Diagnostics, Mannheim) was used to determine serum concentrations of glucose, TC, HDL-C, LDL-C, TG, AST, ALT, GGT, creatinine and uric acid. The following values were defined as high: TC ≥ 200 mg/dL, LDL-C > 130 mg/dL, TG > 150 mg/dL, blood glucose ≥ 100 mg/dL, whereas, the following values were considered low: HDL < 40 mg/dL for males and < 50 mg/dL for females 28–30. Regarding liver enzymes, AST ≥ 30 U/L, ALT ≥ 35 U/L and GGT ≥ 18 U/L were considered high in females, whereas AST ≥ 45 U/L, ALT ≥ 40 U/L and GGT ≥ 28 U/L in males according to the normal upper limit of the ICANS laboratory.

### Dietary intervention

The restricted MD (rMD) was tailored to the patient, considering the energy expenditure related to REE measured by indirect calorimetry and physical activity level. The calorie deficit was 30% of total daily energy requirements. All participants received a restricted MD composed of 40% fat, 15% proteins, and 45% carbohydrates with consumption of traditional Mediterranean foods as olive oil, nuts, and bluefishes, rich of bone-protective nutrients, as calcium vitamin D, high-value proteins, magnesium, zinc. Each patient's allergies, intolerances, and food preferences were also considered. All patients have maintained the vitamin D supplementation, according to the prescribed standard of care. Before the beginning of the dietary protocol, patients and caregivers received preliminary counselling to clarify any doubts and to promote a total understanding of the diet, the attention and time required for meal preparation, food costs, and possible side effects of the diet.

To monitor patients during the follow-up, a periodic interview with specialised dietitians was arranged every month by videoconsultation.

### Statistical analysis

As previously indicated, OI is a rare disease and no study has evaluated our primary outcomes in OI, the standardised formulas for sample size calculation are not applicable. Variables are expressed as means ± SD. Because of the paired data, comparisons of continuous variables, before and after 6 months of intervention, were performed by the paired t test. All *P*-values were two-tailed, and *p* < 0.05 was considered significant. Statistical analysis was performed using IBM SPSS Statistics software version 26.0 for Windows (IBM, Armonk, NY, USA).

## Results

**Fig. 1:**
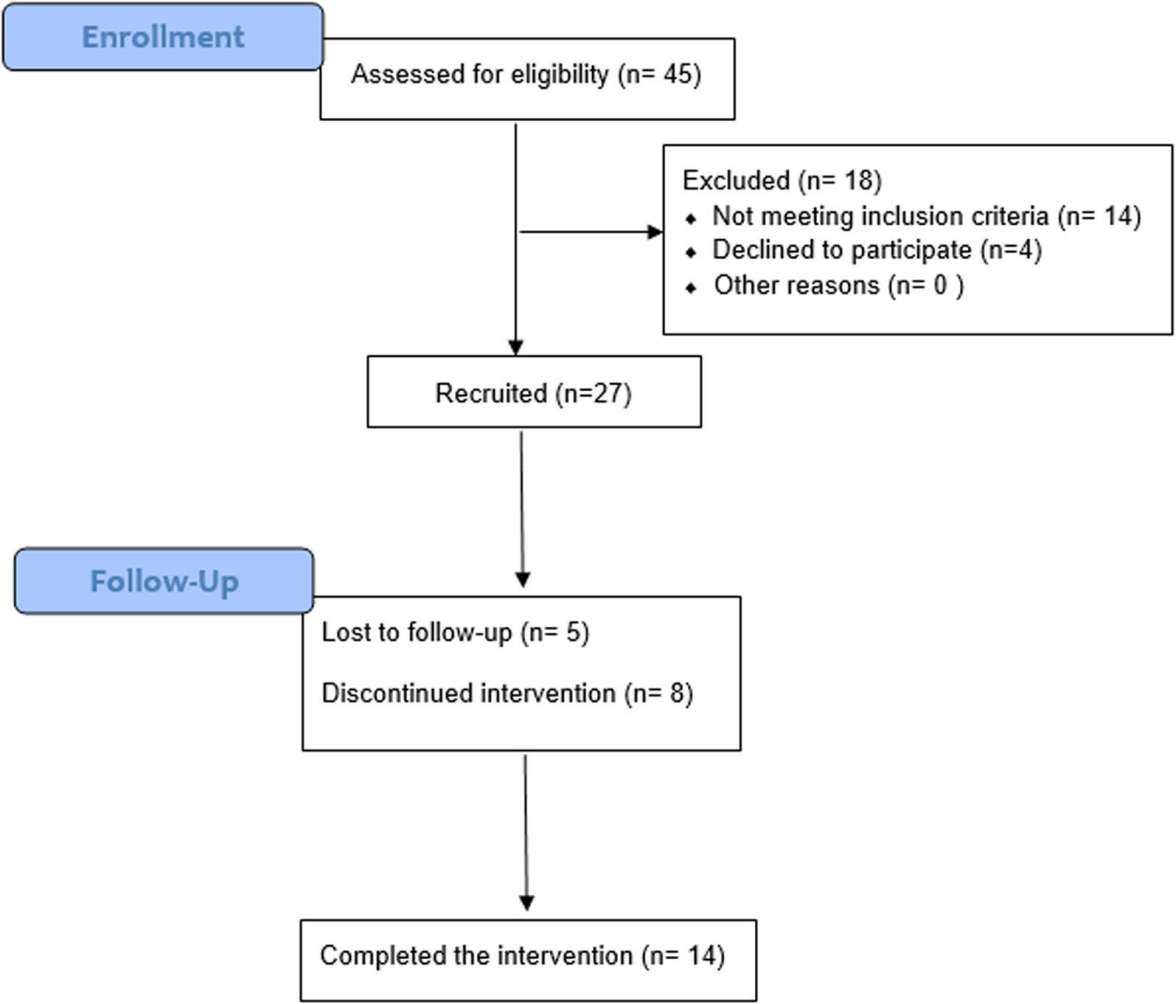
Patients’ flowchart

Twenty-seven patients were recruited (age: 34.6 ± 8.1 years; 71% women). 5 (19%) lost to follow-up and 8 (30%) followed the intervention intermittently, so 14 patients completed the 6-mo protocol (10 affected by OI type III and 4 affected by OI type I). See the flowchart in Fig. 1.

### Dietary intakes

Table 1 shows the daily dietary intakes before and after the beginning of the rMD.

**Table 1:** Daily dietary intake before and after the restricted Mediterranean Diet

	Pre-intervention		Post-intervention		*P* value
	Mean	SD	Mean	SD	
Dietary intake					
MEDAS	6.90	1.60	9.60	1.51	0.032
Kcal/kg BW	31.00	13.21	32.70	10.79	< 0.001
Proteins (g)/kg BW	1.29	0.52	1.51	0.34	0.002
Fat (%)	31.90	5.06	36.70	3.68	0.006
Saturated fat (%)	19.15	2.56	8.91	2.22	< 0.001
Carbohydrates (%)	51.24	6.64	46.89	2.61	0.71
Simple sugars (%)	25.12	8.03	14.89	2.61	< 0.001
Fibre (g)	11.74	6.96	19.49	5.30	0.002
Iron (mg)	5.79	2.45	10.31	2.38	0.003
Calcium (mg)	404.63	306.96	854.11	159.58	0.003
Sodium (mg)	1134.36	753.66	1247.00	610.53	0.253
Magnesium (mg)	78.51	23.05	79.25	19.28	0.297
Zinc (mg)	17.25	44.51	17.08	2.31	0.502
Vitamin D (μg)	1.65	1.47	2.61	1.16	0.070

*MEDAS* MEDiterranean Diet Adherence Score

At baseline, both saturated fats and simple sugars were higher than the recommended [15], and both returned in the normal range according to the diet.

Fibre, iron and calcium intakes were lower than recommended at baseline and they significantly increased after the rMD, doubling the intake consumed and achieving the recommended requirements [15].

As expected, the MEDAS significantly increased, moving from a moderate to a strong adherence. In particular, patients have reported increased consumption of white meat, legumes, fish, nuts, fruits and vegetables.

### Nutritional status

#### Pre-intervention

Baseline nutritional status and biochemical data are summarised in Table 2.

**Table 2:** Nutritional outcomes in 14 adults with OI undergoing restricted Mediterranean Diet

	Pre-intervention		Post-intervention		*P value*
	Mean	SD	Mean	SD	
*Anthropometric parameters*					
BW (kg)	43.59	19.80	40.41	18.85	< 0.001
BMI (kg/m^2^)	30.47	8.09	28.07	7.53	< 0.001
WC (cm)	83.61	18.38	79.61	17.79	< 0.001
NC (cm)	36.52	5.08	34.58	4.63	< 0.001
H/WC	71.88	16.19	68.37	15.61	< 0.001
H/NC	31.62	6.16	29.84	5.32	< 0.001
NC/WC	44.59	5.44	42.47	6.38	0.863
Body Fat (%)	32.92	7.50	29.85	8.44	0.006
AMA (cm^2^)	33.84	12.01	33.24	9.37	0.607
AFA (cm^2^)	29.80	16.12	23.07	15.65	0.011
Resting energy expenditure					
RQ	0.86	0.05	0.85	0.05	0.149
REE (kcal/kg BW)	27.26	6.04	29.20	7.79	< 0.001
REE/pREE %	99.40	14.97	98.90	15.66	0.651
Blood parameters					
Glucose (mg/dL)	86.91	13.17	78.55	15.50	0.003
Uric acid (mg/dL)	4.73	1.02	5.05	1.25	0.107
Urea (mg/dL)	30.64	9.16	29.64	4.78	0.631
Creatinine (mg/dL)	0.39	0.20	0.47	0.20	0.063
TC (mg/dL)	199.45	32.71	184.82	23.59	0.036
HDL-C (mg/dL)	52.00	8.39	58.18	27.45	0.511
TC/HDL	3.91	0.78	3.52	0.90	0.302
LDL-C (mg/dL)	131.55	24.86	119.55	18.84	0.009
TG (mg/dL)	88.91	36.64	87.73	16.70	0.910
GGT	17.64	11.31	15.52	7.04	0.250
AST	19.67	5.29	19.90	5.49	0.805
ALT	20.56	17.33	19.91	12.77	0.762
Vitamin D (ng/mL)	29.19	16.12	30.76	16.05	0.799

*BW* body weight, *BMI* Body Mass Index, *WC* waist circumference, *NC* neck circumference, *H* height, *AMA* arm muscle area, *AFA* arm fat area, *RQ* respiratory quotient, *REE* resting energy expenditure, *pREE* predicted REE using Harris-Benedict equation, *TC* total cholesterol, *HDL-C* high density lipoprotein cholesterol, *LDL-C* low density lipoprotein cholesterol, *TG* triglycerides, *ALT* Alanine Amino Transferase, *AST* Aspartate Amino Transferase, *GGT* γ-glutamyl transferase

8 (57%) patients were overweight, 3 (21%) were affected by obesity type I, 1 (7%) by obesity type II, 2 (14%) were affected by obesity type III. The WC was higher than normal in all these patients.

Regarding biochemical parameters, 3 (21%) patients had hyperglycemia, 1 (7%) hyperuricemia, 5 TC, LDL and TG above the normal range.

#### Post-intervention

The effects of rMD at 6-mo on nutritional status are reported in Table 2*.*

Following the rMD, patients reported a significant reduction in weight and BMI (mean Δweight = − 3.2 ± 2.3 k; *p* < 0.001), accompanied by a significant reduction in NC, WC and AFA and maintenance of AMA. Regarding biochemical parameters, all patients showed blood glucose in the normal range (Δglycemia = − 8.6 ± 7.3 mg/d, *p* = 0.003). Lipid profile values returned to normal levels in 3 out of 5 patients while it also dropped significantly in the remaining 2, who were characterized by abnormal values at baseline (ΔTC = − 14.6 ± 20.1 mg/dL, ΔLDL = − 12.0 ± 12.1 mg/dL, ΔTG = − 11.2 ± 33.7 mg/dL). The patient with hyperuricemia reported normal values at 6-mo.

## Discussion

To our knowledge, this is the first study to investigate the short-term effects of a rMD on nutritional status, in terms of both anthropometric measurements, body composition, resting energy expenditure and biochemical parameters, during a 6-months follow-up in a cohort of adults affected by OI.

A well-tailored restricted Mediterranean diet was effective in the management of nutritional outcomes in overweight and obese patients affected by OI. Excessive body weight may be a factor limiting mobility in adults with OI: a significant decrease in weight, BMI and fat mass at both neck and waist and limb levels occurred in all patients, accompanied by the maintenance of the AMA, an indicator of muscle mass and protein stores [27]. In addition, the diet was also effective in reducing and improving glucose and lipid metabolism parameters in those patients whose values were above normal at baseline.

These results are due to the associated caloric restriction and the Mediterranean dietary pattern with a significant consumption of protective foods, such as legumes, poultry, fish, nuts, fruits and vegetables. These foods provide a high content of fibres, monounsaturated fatty acids and a reduced intake of saturated fatty acids. Furthermore, the MD is characterized by an average intake of seafood, poultry, and dairy products, and low consumption of red meat and sweets. This leads to an improvement in body composition, in terms of maintenance of lean mass and reduction of fat mass, especially at the central level (i.e. neck and waist), associated with increased risk of diseases and reduction of bone mineral content [28, 29].

These findings support the results of Chagas’ et al. on children affected by OI [7]. It further highlights the need for patients with OI of individualised nutritional support to improve body composition, a risk factor for bone fractures in OI, and to potentiate pharmacologic and physical therapies. Moreover, a balanced diet could disrupt the dangerous vicious circle found in OI, in which breathing, sleep and nutritional status are tightly linked, and they might all end up negatively affecting the quality of life [3].

Our sample of OI patients reported a high level of dropout (49% between dropout and intermittent intervention), being in line with those reported regarding dietary interventions aimed at weight loss (57% at 6-months) [30, 31]. Such high dropout is probably due to age < 50 years and psychopathological symptoms, further exacerbated by the disabling disease that dramatically affects the quality of life while putting nutritional issues on the back burner.

The quality of the data collected is one of the study's strengths, as the same specialised operators, thus ensuring less variability, mainly collected all measurements and biochemical assays at the same centre. As far as we know, this is first study that overall assessed the nutritional status of OI adult patients for such a follow-up and that has considered both body composition and REE, in addition to biochemical parameters. Finally, REE has been assessed by indirect calorimetry, the gold standard method for measuring energy needs, and the use of the Harris-Benedict equation to predict REE used in the general population was successful in OI patients reporting a percentage of adherence between measured and predicted REE nearly 100%. However, we are aware of some limitations: a control group was not included in the study and it does not allow discrimination between the effect of overall weight loss and the type of diet (rMD respected to other diet). Another limitation is the small number of patients recruited: OI is a very rare disease, so it is difficult to find a large representative sample. Moreover, the high dropout, especially the discontinuity of the intervention, underscores the need to raise awareness of nutrition as part of the multidisciplinary treatment of disease, strengthening the importance of nutritional counselling in its management. Body composition parameters were measured by a surrogate method as anthropometry and not with the gold standard method dual-energy X-ray absorptiometry (DEXA) [32]. DEXA proved to be a more accurate quantification method because of the physiological and pathological states; however, in the clinical setting, when these methods are not available, alternative methods can be used depending upon the severity of the phenotype of the subject [32]. Finally, no bone-functionality outcomes have been measured to assess diet-related changes, so further studies are needed to test the effectiveness of MD on bone metabolism.

## Conclusion

Here we present the 6-months effects of an rMD in adults with OI. The short-term adherence to rMD had a good safety profile improving anthropometric measurements, body composition, resting energy expenditure and biochemical parameters. We found no evidence of potential adverse effects on the nutritional status of OI. Systematic anthropometric assessment allows early detection of abnormalities in nutritional status in OI that enables specialists to make an effective intervention [33]. MD seems to be greatly appreciated and could increase the supply and synergy between all nutrients involved in bone and collagen metabolism, such as calcium, protein, magnesium, phosphorus, vitamin D, potassium, and fluoride [34].

Our before-and-after comparison study design and the small sample size allowed us to obtain preliminary results, which need to be confirmed in larger long-term clinical studies.

## Data Availability

The data that support the findings of this study are available on 10.5281/zenodo.10618488.

## Abbreviations

rMD: Restricted Mediterranean diet
MD: Mediterranean diet
MEDAS: Mediterranean Diet Adherence Score
TG: Triglycerides
TC: Total cholesterol
LDL-C: Low-density lipoprotein cholesterol
HDL-C: High-density lipoprotein cholesterol
ALT: Alanine aminotransferase
AST: Aspartate aminotransferase
GGT: Gamma-glutamyl transferase
OI: Osteogenesis Imperfecta
BW: Body weight (kg)
BH: Body height (cm)
BMI: Body mass index
NC: Neck circumference
WC: Waist circumference
AMA: Arm Muscular Area
AFA: Arm Fat Area
RQ: Respiratory quotient
pREE: Predicted resting energy expenditure value
REE: Resting energy expenditure
